# Searching for Common Mammalian Retroviruses in Pediatric Idiopathic Diseases

**DOI:** 10.3390/v8030086

**Published:** 2016-03-21

**Authors:** Eric Jeziorski, Vincent Foulongne, Catherine Ludwig, Djamel Louhaem, Michel Rodiere, Marc Sitbon, Valérie Courgnaud

**Affiliations:** 1 Institut de Génétique Moléculaire de Montpellier (IGMM), UMR5535 CNRS, 1919 route de Mende, 34293 Montpellier cedex 5, Université de Montpellier, Place Eugène Bataillon, 34095 Montpellier cedex 5, France; e-jeziorski@chu-montpellier.fr; 2 Hôpital Arnaud de Villeneuve, Service de Pédiatrie générale, infectiologie et immunologie clinique, Centre Hospitalier Régional Universitaire de Montpellier, 371, avenue du Doyen Gaston Giraud, 34295 Montpellier cedex 5, France; c-ludwig@chu-montpellier.fr (C.L.); m-rodiere@chu-montpellier.fr (M.R.); 3 Laboratoire de virologie, Centre Hospitalier Régional Universitaire de Montpellier, 80 avenue A. Fliche, 34295 Montpellier cedex 5, France; v-foulongne@chu-montpellier.fr; 4 Service de Chirurgie Orthopédique, Centre Hospitalier Régional Universitaire de Montpellier, 371 avenue du Doyen Gaston Giraud, 34295 Montpellier cedex 5, France; d-louahemm_sabah@chu-montpellier.fr

**Keywords:** emergent viruses, autoimmune idiopathic disease, pediatric patients, retroviruses, *env* gene, polymerase chain reaction

## Abstract

Mammalian retroviruses cause a variety of diseases in their hosts, including hematological and immunodeficiency disorders. Both human T-cell leukemia (HTLV) and human immunodeficiency (HIV) viruses originated from several independent zoonotic transmissions, indicating that cross-species transmissions from animal to humans may still occur. Thus, as the risk for retroviral transmissions from animals to humans increase, we investigated whether mammalian retroviruses are involved in selected pediatric idiopathic diseases whose symptoms evoke retroviral infections. Blood samples, sera, and synovial fluids, or bone marrow cells were collected from pediatric patients under 18 years of age with different autoimmune idiopathic diseases. Overall, we screened clinical samples from 110 children using sensitive nested and semi-nested PCR strategies targeting *env* genes, and a C-type retrovirus reverse transcriptase (RT) activity kit. All clinical samples were free of retroviral signatures, indicating the unlikelihood of an etiological role of the retroviruses we assessed in the pediatric diseases we tested.

## 1. Introduction

Mammalian retroviruses have been linked to a broad range of neoplastic and non-neoplastic pathologies, including hematological disorders, inflammatory diseases, immunodeficiencies, and neurodegenerative syndromes in various animals. With the striking exception of human T-cell leukemia and human immunodeficiency viruses (HTLV and HIV) for which the first strains were isolated in the early 1980s, the search and involvement of retroviruses in human diseases remain a subject of controversy [1]. Claims that a *xenotropic* murine leukemia virus (XMRV)-related virus could infect humans, for example, have now been discredited, XMRV being a recombined mouse contaminant [2,3,4,5]. Conversely, the presence of a mouse mammary tumor virus (MMTV)-related betaretrovirus in patients with liver autoimmune diseases or breast cancers remains a subject of debate [6,7]. Recently, DNA related to the bovine leukemia virus (BLV) has also been found to be present in human breast tissue samples and to be significantly associated with breast cancer [8,9].

Cross-species transmission of retroviruses among mammals is well established [10,11], and it is thoroughly documented that both HTLV and HIV have emerged from their respective simian counterparts [12,13,14]. Here, we evaluated whether sporadic infectious retrovirus transmissions have accompanied human autoimmune diseases that we selected for clinical manifestations that are reminiscent of retroviral infections. In this context, we focused on pediatric patients, reasoning that strong viremia is likely to accompany pediatric primary infections. Despite the wide array of retroviruses and idiopathic diseases monitored here, we did not find any indication of transmission of the panel of mammalian retroviruses we tested in any of the patient samples.

## 2. Materials and Methods

### 2.1. Pediatric Patients and Sample Collection

We selected a panel of pediatric autoimmune diseases that fulfill either of three criteria: (i) diseases for which an infectious origin was suspected but never proved; (ii) symptoms that are reminiscent of human retroviral infections, such as HIV and HTLV-mediated cytopenia, autoimmune diseases, and vasculitis, all of which occur in HIV-infected patients [15]; and (iii) other pathologies that mimic animal retroviral infections, thrombocytopenia, and autoimmune hemolytic anemia, frequently observed with gammaretrovirus infections [16].

Blood samples, sera, and synovial fluid (in case of arthritis) or bone marrow cells (in case of hematologic diseases or cytopenia) were collected, in accordance with the French Ministry of Health ethical guidelines, from HIV-negative pediatric patients under 18 years of age admitted to the Montpellier University Hospital Center. No particular exposure of the patients to farm animals has been documented; nevertheless, direct contacts to domestic cats and indirect exposure to feral mice are likely. This study was approved by the ethics committee of the Délégation Régionale à la Recherche Clinique et à l'Innovation Languedoc—Roussillon—Montpellier (N° DC-2009-1052). All patients, or their legal representatives, gave their written informed consent.

### 2.2. Mammalian Retrovirus Selection

We chose to monitor retroviruses that are able to infect human cell lines *in vitro.* We restricted our search to *env* sequences related to selected retroviruses and thus looked for potential infections with: simian T-cell leukemia virus (STLV)or human T-cell leukemia virus (HTLV) and BLV deltaretroviruses; MMTV, betaretroviruses that have been described in breast cancers and autoimmune cholangitis [1,7]; the Mason-Pfizer monkey virus (MPMV), a betaretrovirus that causes cytopenia in macaques; and members of the gammaretrovirus genus, including the gibbon ape leukemia virus (GaLV), feline leukemia viruses (FeLV) and the infectious porcine endogenous retroviruses (PERV), which all use different receptors to enter human cells. BLV, FeLV, PERV, and MMTV were of special interest because of the close proximity and frequent contacts of their natural hosts with humans, and the xenotransplantation procedures using pig materials [10,17].

### 2.3. Retrovirus Detection

We monitored two markers of retroviral presence, reverse transcriptase (RT) activity, and proviral DNA. RT in sera and synovial fluid cells was assessed according to the manufacturer’s instructions with the highly sensitive non-radioactive ELISA C-type RT activity kit (Cavidi, Uppsala, Sweden) that efficiently targets gammaretroviruses, the prototype of which are murine leukemia viruses, including those that can infect human cells (xenotropic, polytropic, and amphotropic MLV). Sensitivity of all RT assays was assessed with the kit sample controls, with the expected range of sensitivity. DNA extraction was performed using the QIAamp blood kit (Qiagen, Courtaboeuf, France) as previously described [4]. DNA concentrations were determined with a Nanodrop ND-1000 spectrophotometer. To verify the quality of DNA extracts, a region of Glyceraldehyde 3-phosphate dehydrogenase (GAPDH) was amplified using single-round PCR.

In addition, bacterial exploration with direct examination and culture was performed in all synovial fluid samples with no bacterial agent found. Briefly, all synovial fluid samples were inoculated onto standard agar for aerobic and anaerobic cultures during five days, and into Schaedler Broth up to 15 days, in accordance with the European guidelines [18] ,and a smear was performed for Gram staining.

Using a multiple envelope alignment of available strains for each class of retrovirus, we were able to identify short highly conserved motifs, specific for each class, and scattered within the highly variable *env* region. We therefore designed specific primers that matched these highly conserved motifs, which are present in all members of a given class of *env* receptor-binding domain (RBD). These sets of primers distinguished BLV, FeLV, PERV, GaLV, MMTV, or MPMV while encompassing all members of each class (Table 1). This strategy, with which we have identified new HTLV and STLV variants [19], maintained the potential to isolate new retroviruses or variants that are likely to share those highly conserved short motifs.

PCR was performed based on a sensitive semi-nested assay initially developed to detect all known HTLV and STLV types (1–4) [19]. Anchoring PCR on conserved motifs within the RBD, which is the most variable region of *env*, thus selectively enhanced detection of exogenous infections. To ensure the absence of detection of human endogenous retroviral DNA sequences, primer specificity was verified *in silico* [20]. and confirmed experimentally using PCR performed on human peripheral blood mononuclear cell (PBMC) DNA from different individuals. Our PCR sensitivity was estimated either with *env*-containing plasmids diluted in PBMC DNA or, in the case of MPMV, with HEK-293T-infected cells harboring at least 1 viral copy per cell. The threshold sensitivity of all our PCR conditions corresponded to 10 to 20 copies per reaction in 500 ng of human PBMC DNA.

## 3. Results and Discussion

We analyzed 120 samples for RT activity and 64 samples via PCR using High Fidelity Platinum® *Taq* DNA Polymerase (Life Technologies, Carlsbad, CA, USA) together, samples were obtained from a group of 110 children. As summarized in Table 2, both *env* amplification and RT activity detection were free of retroviral signatures.

Despite the fact that we could not formally exclude traces of retroviral presence, our results strongly indicate that there was no link between these pathologies and infection with the wide range of retroviruses related to those we targeted.

The search for new retroviruses using PCR in samples from adult patients with a variety of diseases has led to many controversial results, in part due to contamination or background amplification of endogenous sequences [1]. For some pediatric immune-mediated diseases, involvement of retroviruses has also been suspected, as in, for example, the search of RT activity in samples from children with Kawasaki disease, which led again to contradictory results [21,22,23]. However, to our knowledge, our study is the first one that specifically investigated several retroviral infections as possible etiologic agents in a panel of pediatric autoimmune diseases.

Here, we used a new *env*-specific approach directed against retroviruses that we selected for their potential transmission to humans and in association with pediatric diseases. Efficient detection was achieved with nested PCR, or semi-nested PCR in the case of PTLV detection, while specificity was ensured by selecting an arrangement of consensus motifs within the Env RBD variable domains. We previously described the effectiveness of this method by detecting new HTLV-1/STLV-1 variants [19] or HTLV-2/4 variants (unpublished results). While we are confident that the pediatric disease samples we tested did not contain retroviruses that harbor the targeted *env* sequences, we cannot formally eliminate the potential presence of infectious retroviruses that would harbor widely divergent *env*. Obviously, application of next-generation sequencing (NGS) techniques would greatly improve the monitoring for retroviral infections in human autoimmune diseases once reliable distinctions of potentially infectious exogenous retroviruses from human endogenous retroviral sequences can be made with deep sequencing [24].

## Figures and Tables

**Table 1:** PCR primer sets used to amplify *env*-specific sequences from the indicated retroviruses.

Virus	Primer Sequence 5’ ----> 3’	Genbank Accession Number
BLV	F1: CCAGACTTGGAGATGCTCCCTG (4707-4728) ^1^ R1: GCACGGGCCTTCCCAGATTGTG (6443-6466) F2: GATGACAGCATATAACCAAGAGG (4749-4771) R2: GCCCAAATCTTCTGGGTCAACA (5347-5368)	AF033818
FeLV	F1: ATTTTGCAGTTCACCCAGAAGG (6552-6573) R1: TATACCGTACAGGATATGACCC (6619-6640) F2: GGTCTTAGGAACAGTCCCTATGC (6619-6640) R2: GGGGAGGGTTTGTTTGGTCGCTG (7069-7091)	NC_001940
PERV	F1: CTGAAAATCCCCTTAAGCTTCG (5542-5563) R1: ACATTGAATTTCCCTCCTCTAGCC (6504-6527) F2: TCCATCGCGTGGTTCCTTACTC (5566-5587) R2: GCTAAGCAAAGCCAACAAGAAG (6455-6476)	EU789636.1
GaLV	F1: TGGCAGGTACTGTCCCAAACTGG (5720-5742) R1: GTTAGGAAGGCCCCCTGCAC (6557-6577) F2: TGGGGAGCCGTAGGGTGCAGCTAC (5926-5950) R2: GGGAGGCGGAGTGTTTAGGGTAAC (6506-6529)	NC_001885.2
MMTV	F1: ACTTCACAAACTCCCCAAACCTC (6774-6796) R1: ACAGGGGGAGTATAATTTCCAAG (7462-7440) F2: GTGGGATGGGGAAGTACAGACC (6897-7918) R2: AGGAGAATTCTCCCAGAACCAAG(7519-7543)	AF228552.1
MPMV	F1: CTTGTCACTGTGCTGGAGGATATG (5742-7765) R1: GATCTCCTGACTGTAAGCACAGCC (6363-6387) F2: TCGATCTTTTCTTTACCACGGGC (6266-6282) R2: AAACTCCCTAAAATGGCAGTGTG (5832-5855)	NC_001550.1

^1^: Numbers in parentheses indicate the position of each primer as in the corresponding GenBank sequence. BLV: bovine leukemia virus; FeLV: feline leukemia virus; PERV: porcine endogenous retrovirus; GaLV: gibbon ape leukemia virus; MMTV: mouse mammary tumor virus; MPMV: Mason-Pfizer monkey virus.

**Table 2:** Patient characteristics and processing of pediatric samples. PCR analyses were performed with the primer sets shown in Table 1, designed specifically to amplify *env* sequences from BLV, FeLV, PERV, GaLV, MMTV, MMPV, and PTLV. MLV, FeLV, PERV or spumavirus presence was assayed by RT assay.

Pathologies	Patients (No.)	Age Range	Sample	PCR Analyses (Sample No.)	RT Assay (Sample No.)
Acute thrombocytopenia ^1^	35	2 mos–17 yrs	Whole blood Serum	10 NA	NA 31
			Bone marrow	3	NA
Autoimmune hemolytic anemia	3	4–16 yrs	Whole blood Serum	3 NA	NA 3
			Bone marrow	1	NA
Juvenile idiopathic arthritis ^2^	59	1–15 yrs	Whole blood	15	NA
			Serum	NA	24
			Bone marrow	1	NA
			Synovial fluid	28	49
Henoch-Schönlein disease	9	2–13 yrs	Whole blood	3	NA
			Serum	NA	9
Kawasaki disease	4	7 mos–3 yrs	Serum	NA	4
Total	110			64	120

^1^: Including 29 idiopathic thrombocytopenic purpura, 6 documented infectious-associated thrombocytopenia (1 enterovirus, 2 cytomegalovirus, 2 Epstein Barr Virus, 1 parvovirus); ^2^: Including 41 oligoarticular, 14 polyarticular, 3 HLAB27-positive, 2 systemic onset; RT: Reverse transcriptase; mos: months; yrs: years.
